# Kojic acid repurposing as a pancreatic lipase inhibitor and the optimization of its production from a local *Aspergillus oryzae* soil isolate

**DOI:** 10.1186/s12896-020-00644-9

**Published:** 2020-10-02

**Authors:** Sarah Mohamed El-Korany, Omneya Mohamed Helmy, Ali Mahmoud El-Halawany, Yasser El-Mohammadi Ragab, Hamdallah Hafez Zedan

**Affiliations:** 1 Department of Microbiology and Immunology, Faculty of Pharmacy, Cairo University, Cairo, Egypt; 2 Department of Pharmacognosy, Faculty of Pharmacy, Cairo University, Cairo, Egypt

**Keywords:** Anti-obesity, Pancreatic lipase inhibitor, Soil fungi, *Aspergillus oryzae*, Kojic acid, Orlistat, Anti-triglyceride

## Abstract

**Background:**

Obesity and its related diseases are increasing worldwide. One of the best therapeutic strategies for obesity management is through the inhibition of pancreatic lipase (PL) enzyme. So far orlistat is the only FDA approved PL inhibitor, but with unpleasant side effects. New efficacious anti-obesity drugs are needed to achieve a successful reduction in the incidence and prevalence of obesity. Many microbial metabolites have PL inhibitory activity. Screening soil inhabitants for PL inhibitors could help in increasing the available anti-obesity drugs. We aimed to isolate and identify alternative PL inhibitors from soil flora.

**Results:**

We screened the crude mycelial methanolic extracts of 39 soil samples for PL inhibitory activity by the quantitative lipase colorimetric assay, using the substrate *p*-nitrophenyl palmitate and orlistat as positive control. AspsarO, a PL inhibitor producer, was isolated from an agricultural field soil in Giza, Egypt. It was identified as *Aspergillus oryzae* using colony morphology, microscopical characteristics, *18S* rDNA sequencing, and molecular phylogeny. Increasing the PL inhibitor activity, in AspsarO cultures, from 25.9 ± 2% to 61.4 ± 1.8% was achieved by optimizing the fermentation process using a Placket–Burman design. The dried 100% methanolic fraction of the AspsarO culture had an IC_50_ of 7.48 μg/ml compared to 3.72 μg/ml for orlistat. It decreased the percent weight gain, significantly reduced the food intake and serum triglycerides levels in high-fat diet-fed Sprague–Dawley rats. Kojic acid, the active metabolite, was identified using several biological guided chromatographic and ^1^H and ^13^C NMR techniques and had an IC_50_ of 6.62 μg/ml. Docking pattern attributed this effect to the interaction of kojic acid with the key amino acids (Lys80, Trp252, and Asn84) in PL enzyme binding site.

**Conclusion:**

Combining the results of the induced obesity animal model, in silico molecular docking and the lipase inhibitory assay, suggests that kojic acid can be a new therapeutic option for obesity management. Besides, it can lower serum triglycerides in obese patients.

## Background

Obesity, a metabolic disorder characterized by the accumulation of lipids, poses a risk to human health [1]. It is associated with serious health conditions like insulin resistance, diabetes mellitus, cardiovascular diseases, and certain types of cancer, including endometrial, breast, ovarian, prostate, liver, gallbladder, kidney, and colon cancer [2, 3]. The World Health Organization (WHO) reported that 2.8 million people die annually because of obesity-related diseases [4]. There are more than 1.5 billion overweight adults worldwide, and this number is expected to rise to 3.3 billion by 2030 [5]. Among the 20 most populous countries, the highest level of adult obesity (35.3%) was recorded in Egypt in 2015 [6].

Pancreatic lipase (PL) is the key enzyme in lipid metabolism. Its inhibition alters the absorption of the ingested triglycerides and is thus considered one of the major targets in obesity management [7]. Orlistat is the only FDA approved PL inhibitor for obesity treatment [8]; it is a saturated derivative of lipstatin, a potential natural PL inhibitor, isolated from the actinobacterium *Streptomyces toxytricini* [9]. However, it has many side effects including oily stools, flatulence, fecal urgency, and abdominal cramps [10]. Hepatotoxicity, the formation of gall and kidney stones, and acute pancreatitis are severe adverse effects occurring due to the long-term administration of orlistat [8]. These side effects have motivated researchers to explore new alternative sources for pancreatic lipase inhibitors, such as plants, bacterial, fungal, and marine species [7, 8, 11, 12], or synthesize completely synthetic PL inhibitors. Cetilistat (ATL-962) is a new synthetic PL inhibitor that had completed phase III clinical trials but is not yet approved [13].

Fungi are considered microbial cell factories that can produce various bioactive agents, including antitumor, antibacterial, antifungal, antiviral, and enzyme inhibitor compounds [14]. Aspergilli are ubiquitous filamentous fungi, known to secrete antibiotics, mycotoxins, immune-suppressants, and cholesterol-lowering agents [15–17]. Kojic acid (5-hydroxy-2-hydroxymethylgamma-pyrone, KA) is a major secondary metabolite of *Aspergillus oryzae*, *Aspergillus flavus, Aspergillus tamarii*, and *Penicillium* species [18, 19]. Because of its biocompatibility, kojic acid has many medical applications. These include antimicrobial, antiviral, antitumor, antidiabetic, anticancer, antiparasitic, antioxidant, anti-proliferative, and anti-inflammatory activities [20]. KA also acts as a UV protector and suppressor of skin hyper-pigmentation owing to its tyrosinase inhibitory activity [21].

Egyptian soil is an under-explored resource for PL inhibitors. We aimed to isolate and identify soil fungal lipase inhibitor producer(s) and test the lipase inhibitor effect by in vitro and in vivo assays. The bioactive compound, from the most potent isolate, was further purified and characterized.

## Results

### Screening soil samples for possible fungal PL inhibitory effect

The methanolic extracts of 39 mycelial mats, resulting from culturing soil samples in starch casein broth, were tested for PL inhibitory activity by the quantitative lipase colorimetric assay, using the substrate p-nitrophenyl palmitate and orlistat as a positive control. Fifteen soil samples showed PL inhibitory activity; all of which were agriculture field or garden soils (Table 1).

**Table 1:** Screening crude soil mycelial extracts for PL inhibitory activity

Sample number	Type of soil	Area of collection	% PL inhibition
S1	Agriculture field	Monofiya	0
S2	Agriculture field	Monofiya	13
S3	Agriculture field	Monofiya	1.3
S4	Agriculture field	Monofiya	10.1
S5	Agriculture field	Monofiya	4.7
S6	Agriculture field	Monofiya	17.2
S7	Garden	Cairo	6
S8	Garden	Cairo	6.3
S9	Agriculture field	Monofiya	0
S10	Agriculture field	Monofiya	0
S11	Garden	Elein Elsokhna	13.3
S12	Garden	Elein Elsokhna	0
S13	Garden	Elein Elsokhna	0
S14	Garden	Elein Elsokhna	0
S15	Garden	Elein Elsokhna	0
S16	Sandy	Elein Elsokhna	0
S17	Sandy	Elein Elsokhna	0
S18	Sandy	Elein Elsokhna	0
S19	Sandy	Elein Elsokhna	0
S20	Soil (sewer)	Cairo, magraeloyon	0
S21	Soil (sewer)	Cairo, magraeloyon	0
S22	Soil (sewer)	Cairo, magraeloyon	0
S23	Soil (sewer)	Cairo, magraeloyon	13.3
S24	Nile river bank	Cairo	0
S25	Nile river bank	Cairo	0
S26	Nile river bank	Cairo, Helwan	0
S27	Nile river bank	Cairo, Helwan	0
S28	Garden	Kanater	0
S29	Garden	Warraa	0
S30	Garden	Monofiya	0
S31	Garden	Monofiya	0
S32	Garden	Monofiya	0
S33	Garden	Monofiya	16.3
S34	Agriculture field	Giza, Saftlabban	46
S35	Agriculture field	Giza, Saftlabban	0
S36	Agriculture field	Giza, Saftlabban	12
S37	Agriculture field	Giza, Saftlabban	2.8
S38	Garden	Cairo, Nasr city	11.8
S39	Garden	Cairo, Garden city	8.9

### Isolation and identification of PL inhibitor producers from soil sample S34

We isolated three different fungal isolates from the soil sample, S34, recording the highest percentage of PL inhibition. We tested the methanolic extract of each of them for PL inhibitory activity. The isolate (AspsarO) showed a percentage PL inhibition of 35.5% ± 3.0 compared to orlistat (41% ± 2.45) with no significant difference between them (*p* > 0.05). For further confirmation of PL inhibition, the 100% methanolic fraction of AspsarO extract was retested by a fluorometric assay using the substrate 4-methyl-umbelliferyl butyrate. There was no significant difference between the percentage of PL inhibition by AspsarO (20% ± 4.583) and orlistat (19.67% ± 4.485) (*p* > 0.05). These findings suggested that AspsarO is a possible PL inhibitor producer.

**Fig. 1:**
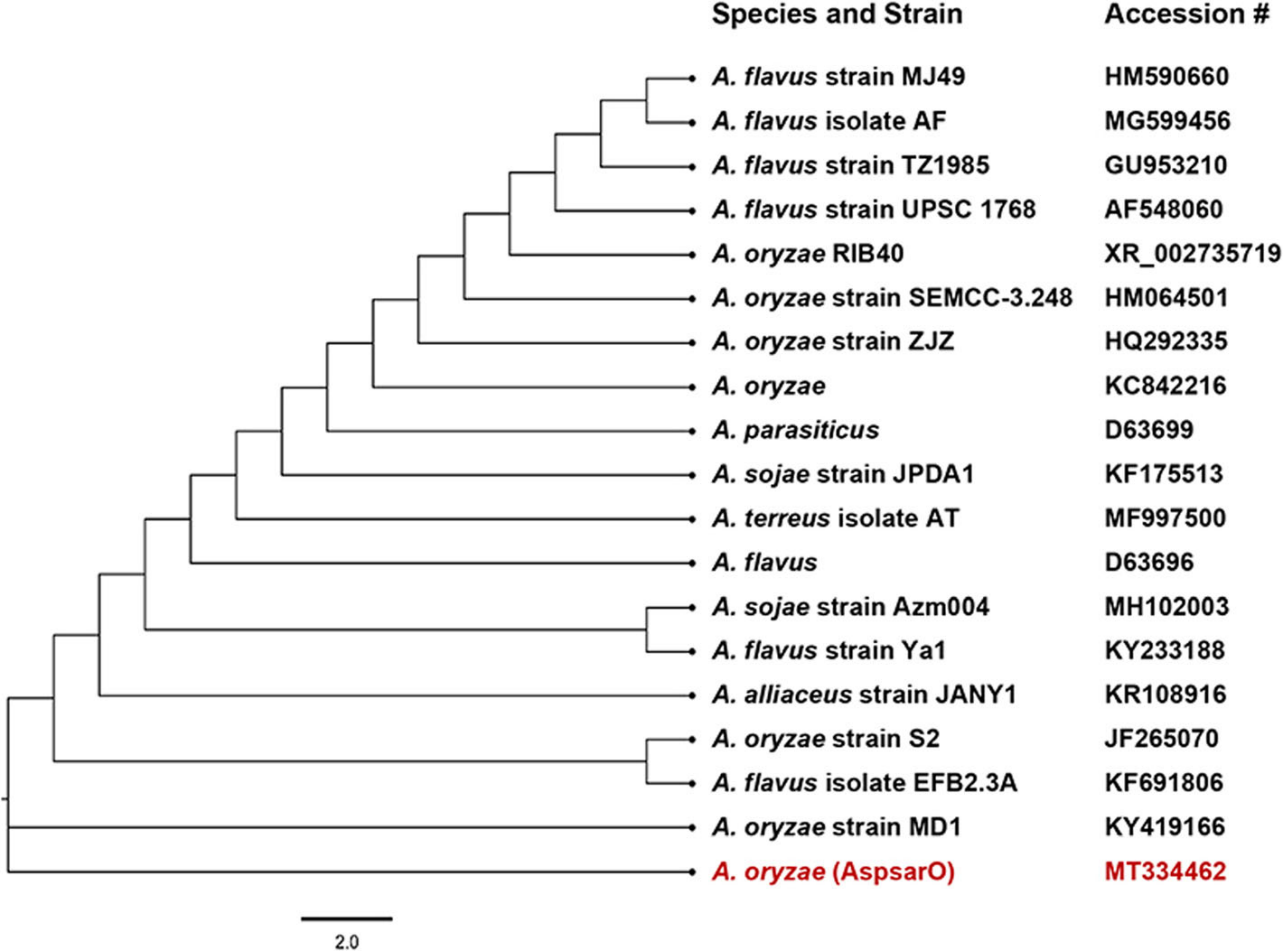
Phylogenetic tree for AspsarO, based on *18S* rDNA sequence analysis using the maximum composite likelihood method. The tree was constructed using MEGA-X, where the evolution distances from hypothetical ancestors are represented by nodes between the AspsarO isolate and closely related Aspergilli. AspsarO partial *18S* rDNA was deposited in Genbank under accession no. MT334462

To identify AspsarO, the macromorphological characteristics of its colonies on PDA, CYA, and MEA were examined. On both PDA and MEA, AspsarO colonies were yellowish-green with white mycelia at the edges, while the colonies on CYA were yellow at the center with white mycelia at the edges. The conidia were rough and did not produce any exudates or soluble pigments in the tested media (Supplementary Figure 1). The micromorphological features of AspsarO, conidia heads, and conidia are shown in Supplementary Figure 2. These findings suggest that AspsarO belonged to the *Aspergillus* species. Also, *18S* rDNA sequencing was performed for the molecular identification of AspsarO. The obtained sequence was blasted against the nucleotide database using blastn tool, of the US National Centre for Biotechnology Information (NCBI), and showed 100% identity to *Aspergillus oryzae* RIB40 DNA, chromosome 7 (NC_036441.1). It was deposited in GenBank under GenBank accession no. (MT334462). We constructed a phylogenetic tree based on *18S* rDNA sequence of AspsarO and the closely related species using MEGA-X (Fig. 1) and this further confirmed the identification.

### Optimization of lipase inhibitor production by AspsarO

We used sequential optimization approaches. A one-variable-at-a time method was used for the determination of the optimum factors for PL inhibitor production by AspsarO. This was followed by testing  different combinations of factors using a multifactorial design.

### Factors affecting PL inhibitor production

**Fig. 2:**
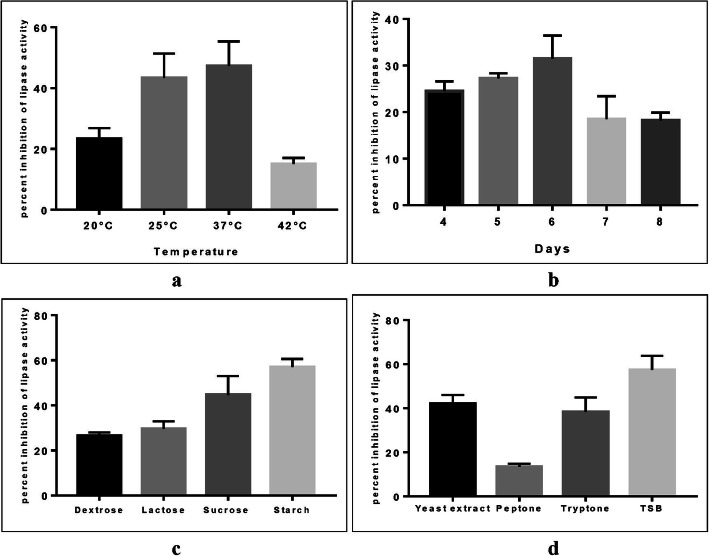
Optimization of lipase inhibitor production by AspsarO where: **a** Different incubation temperatures in PDB for 6 days. **b** Incubation time at 30 °C in PDB. **c** Different carbon sources in potato broth cultures at 30 °C for 6 days. **d** Different nitrogen sources in PDB at 30 °C for 6 days. The data presented are the mean of three independent experiments, and the error bars represent the standard deviation (SD)

The percentage PL inhibition was determined following the incubation of AspsarO under different incubation temperatures and time, using alternative carbon sources and different nitrogen supplements in the fermentation medium. PL inhibitor production increased by increasing the incubation temperature with the highest yield recorded at 37 °C (Fig. 2-a). Also, it increased by prolonging the incubation time with the optimum production recorded on day six (Fig. 2-b). Replacing dextrose in PDB with starch almost doubled the PL inhibitor activity (Fig. 2-c); PDB with tryptic soy broth produced approximately 1.5 times the amount of PL inhibitor compared to PDB (Fig. 2-d).

### Multi-factorial design

Lipase inhibitor production was optimized by testing a combination of factors using a multi-factorial design (Plackett-Burman design). The standardized effect of each factor (E-value) was calculated using Minitab software (version 18), and the E-value of the measured response was recorded as percentage PL inhibition (Table 2).

**Table 2:** Placket-Burman design matrix for determination of optimum conditions for PL inhibitor production by AspsarO

Runs	Factors				Response^c^
	Incubation Temperature (°C)	Production time in Days	Carbon source replacing dextrose in PDB	Nitrogen source	% PL inhibition ± SD
1	25	5	sucrose	YE ^a^	44.8 ± 3.3
2	37	5	sucrose	YE	48.0 ± 2.6
3	25	6	sucrose	YE	50.0 ± 2.6
4	37	6	sucrose	YE	53.1 ± 4.1
5	25	5	starch	YE	47.9 ± 1.4
6	37	5	starch	YE	46.1 ± 1.1
7	25	6	starch	YE	56.4 ± 0.9
8	37	6	starch	YE	48.0 ± 1.2
9	25	5	sucrose	TSB ^b^	48.2 ± 3.3
10	37	5	sucrose	TSB	38.9 ± 0.9
11	25	6	sucrose	TSB	51.7 ± 5.7
12	37	6	sucrose	TSB	56.6 ± 2.0
13	25	5	starch	TSB	25.9 ± 2.4
14	37	5	starch	TSB	51.2 ± 0.7
15	25	6	starch	TSB	57.5 ± 2.3
16	37	6	starch	TSB	61.4 ± 1.8
17	25	5	–	–	43.3 ± 8.0

^a^ yeast extract, ^b^ tryptic soy broth. ^c^All runs were performed in triplicates; the response is represented as the mean of three independent experiments ± the standard deviation

**Fig. 3:**
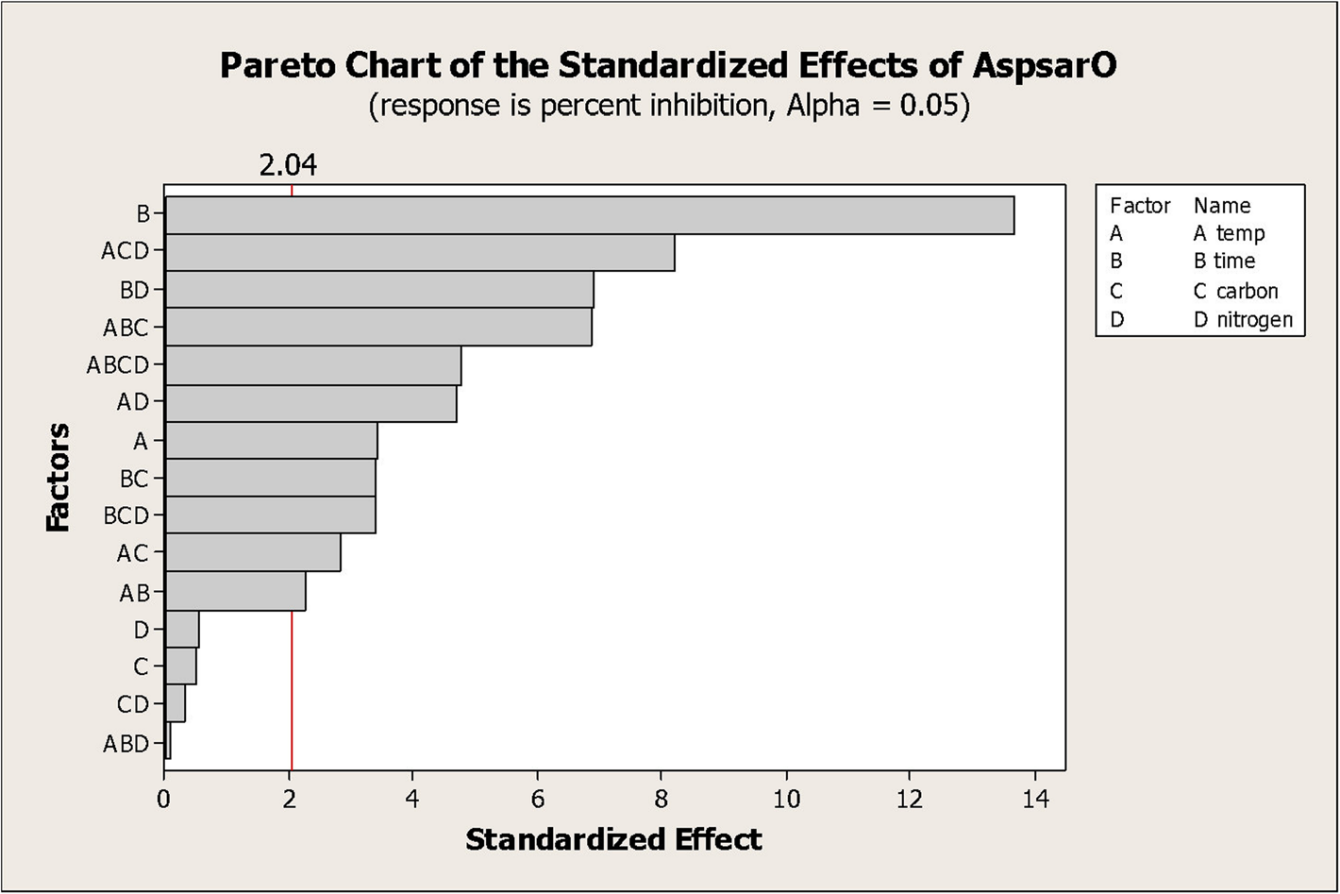
Pareto chart ranking the standardized effects of different variables on lipase inhibitory activity. The vertical line in chart represents a reference line, any factor that exceeds this line is of significant effect at alpha = 0.05 (significance level). Incubation time and temperature significantly affected the inhibitor production

**Fig. 4:**
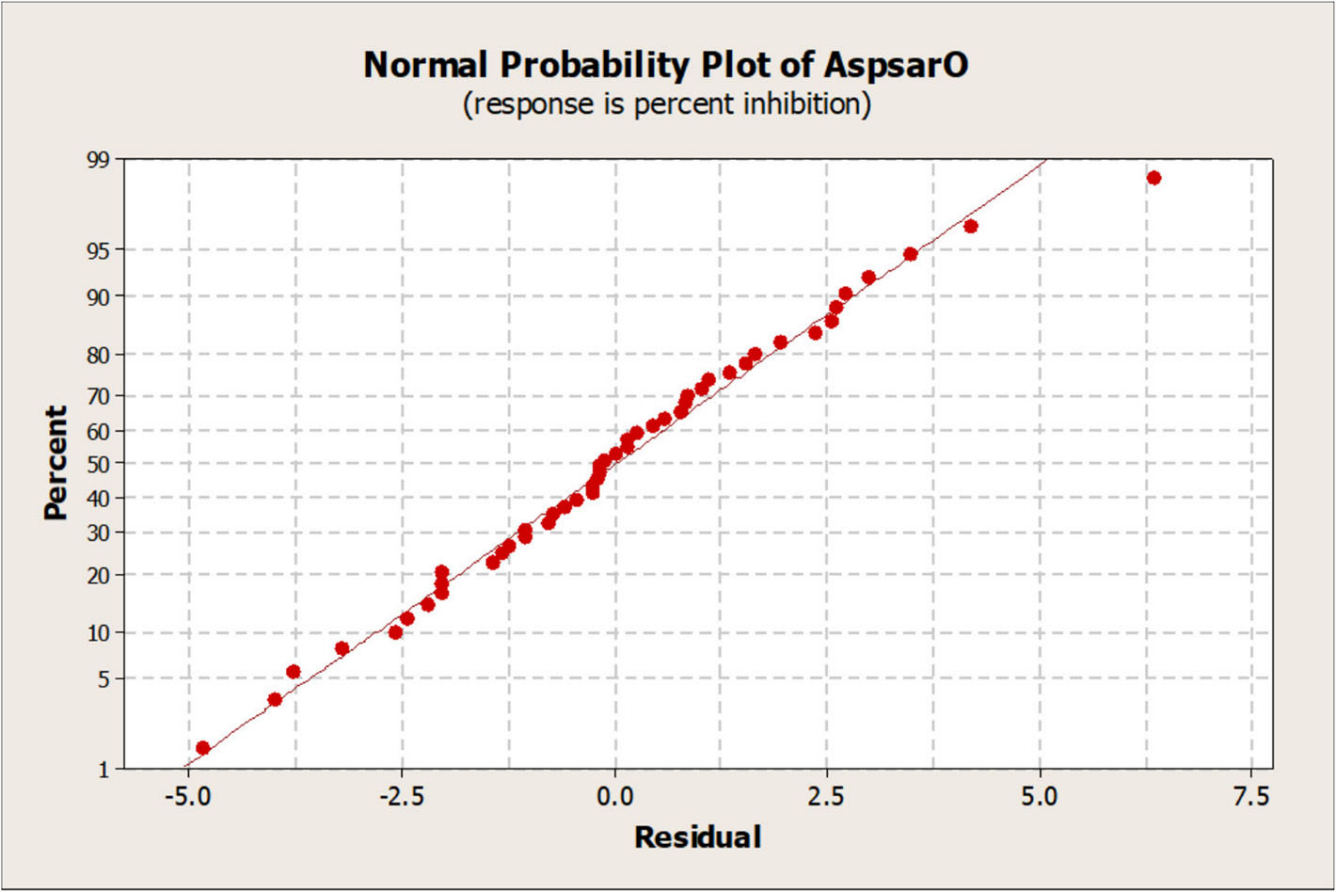
Normal probability plot of residuals by AspsarO. The straight line in the graph represents the mathematical regression equation (which determines the expected data), while the dots in the plots represent the actual observed data. As shown in figure, the dots generally formed a line consistent with regression line, therefore the residuals (the difference between observed data and expected data) are considered normally distributed

**Fig. 5:**
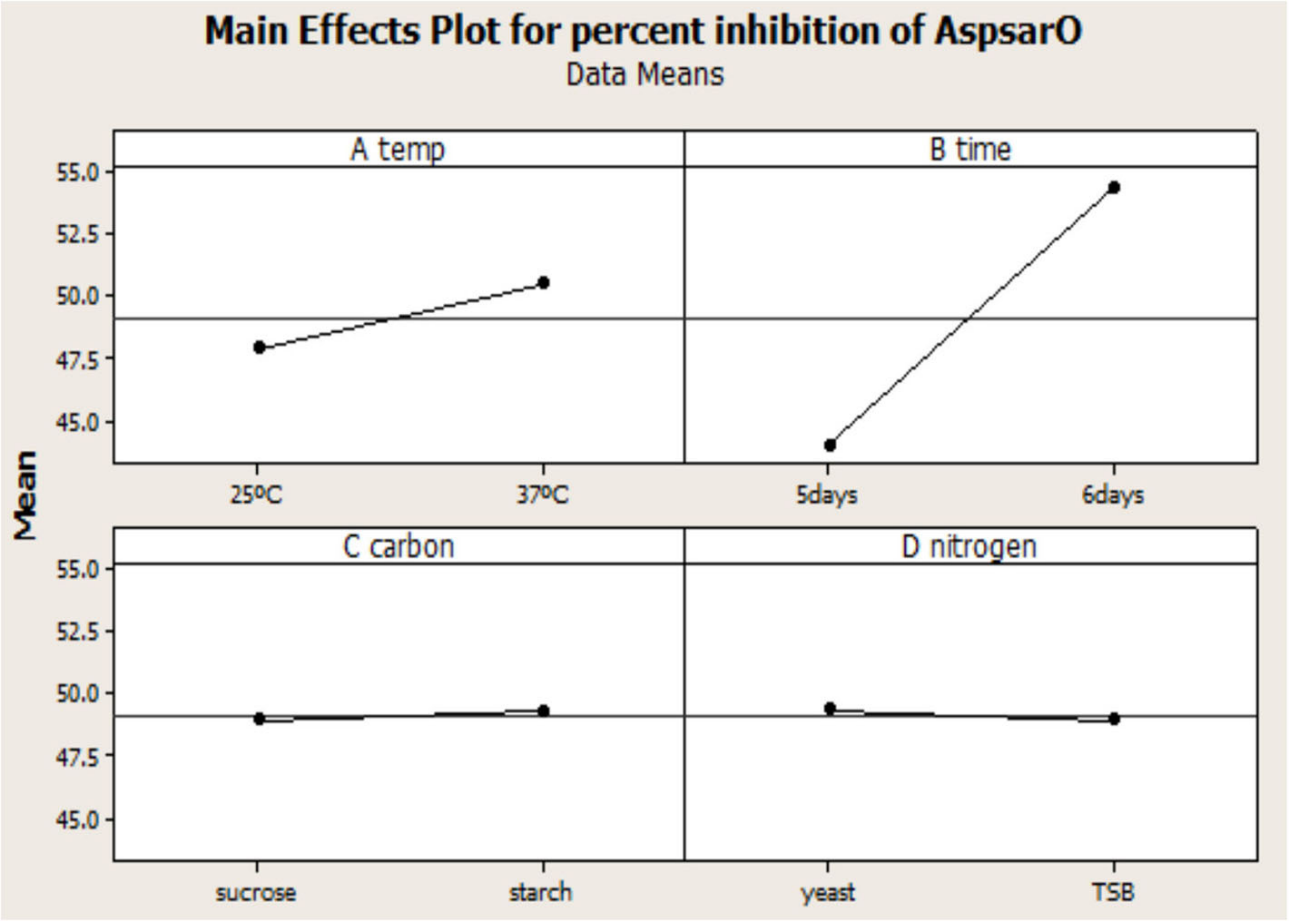
Main effect plot comparing the magnitude of the main effects of factors. The steeper the slope of the line is, the greater the magnitude of the main effect. When the line is horizontal (parallel to the x-axis), no main effect is present. Incubation time and temperature affected lipase inhibitor production

The value of the predicted determination coefficient (R^2^_Pred_) was 0.84, and the adjusted determination coefficient (R^2^_Adj_) was 0.90. The Pareto chart showed the standardized effect, magnitude and significance of each factor (Fig. 3). Percentage PL inhibition ranged from 25.9 to 61.4%, with the highest percentage recorded by increasing the incubation time and temperature. Normal probability plot of residuals examined the goodness of the model fit (Fig. 4), and the residuals were normally distributed. The main effect plot determined the main effects of all factors; it revealed that PL inhibitor production was influenced by both incubation time and temperature (Fig. 5). Thus, the incubation time and temperature significantly affect the response, while the carbon and nitrogen sources are non-significant factors.

### Purification and characterization of the active PL inhibitor

AspsarO was inoculated in eight liters potato starch broth supplemented with tryptic soy broth and incubated at 37 °C for 6 days for maximum PL inhibitor production. The biomass was separated from the cultured broth, which was subjected to fractionation using methylene chloride. The remaining aqueous layer was concentrated under vacuum and applied to a DiAION HP-20 column.

Percentage of PL inhibition by the AspsarO fractions were 23, 0, 36, and 43% for the methylene chloride, aqueous, 50, and 100% methanolic fractions, respectively. Purification, of the 100% methanolic fraction, was performed using bio-guided chromatographic techniques. This resulted in the isolation of kojic acid as the main constituent of the fraction. The identity of the compound (off-white amorphous powder) was confirmed by ^1^H NMR spectral data (CD_3_OD, 400 MHz); 4.35 (2H, s, H-7), 6.39 (1H, s, H3), 7.85 (1H, s,H6) and ^13^C NMR spectral data (CD_3_OD, 100 MHz); 59.8 (C7), 110.1 (C3), 139.6 (C6), 146.0 (C5), 170.0 (C2), 175.5 (C4), and by comparison to literature [22].

### Molecular docking study

**Fig. 6:**
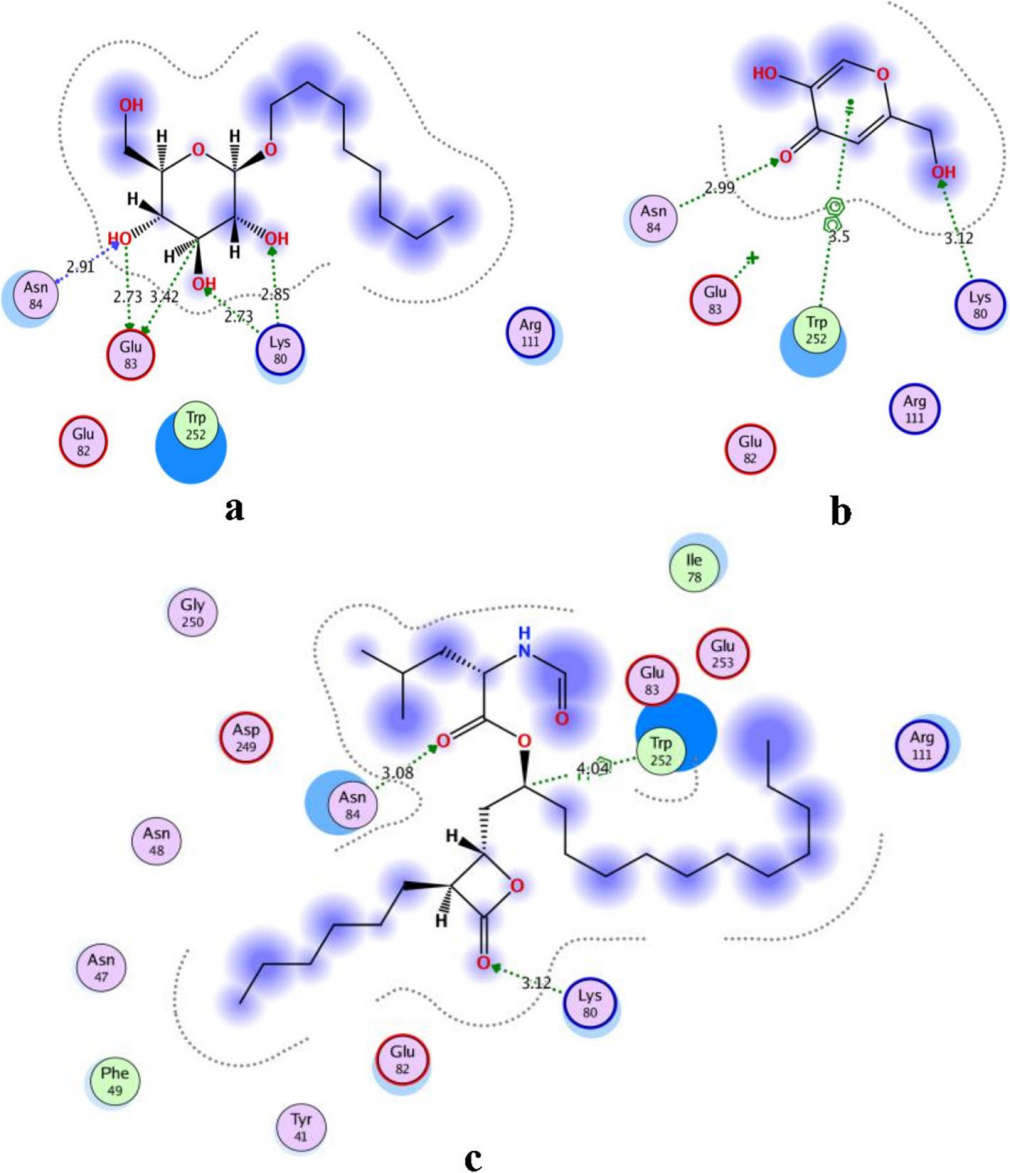
2D docking pose interaction diagrams with the key amino acids in human PL binding site. **a** BOG, **b** Kojic acid and **c** Orlistat

**Fig. 7:**
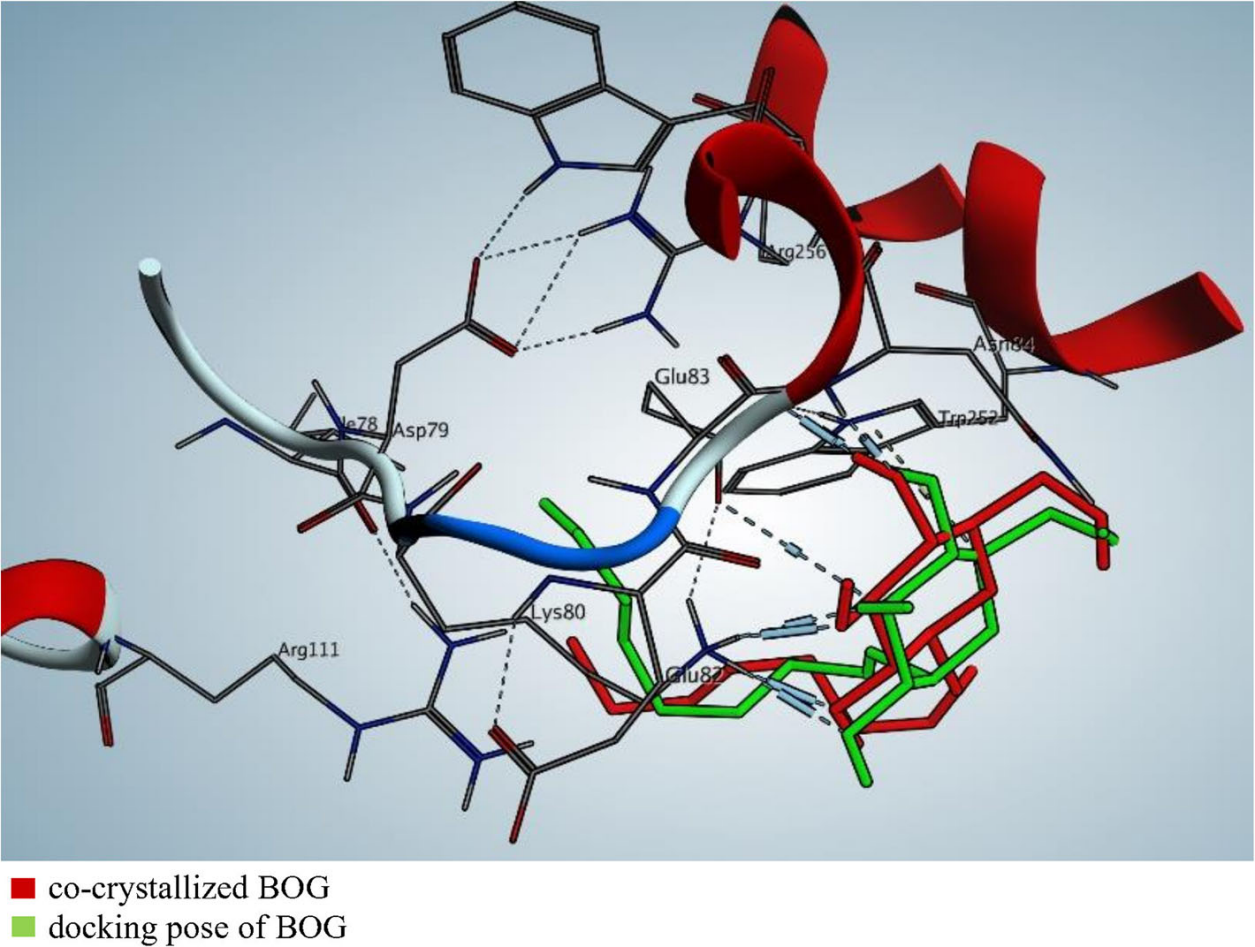
3D superimposition of the co-crystallized and the docking pose of BOG in PL binding site. The superimposition of the co-crystallized BOG in pancreatic lipase binding site is represented in red color and its docking pose is represented in green color; the superimposition was with RMSD of 2.0834 A°

The binding modes and affinities of kojic acid, within the binding site of the human pancreatic lipase enzyme, was predicted using molecular docking. Self-docking of the co-crystallized ligand, B-Octylglucoside (BOG), of PL enzyme in the vicinity of its binding site was validated in docking setup (Fig. 6-a). BOG docking pose interactions with the key amino acids (Lys80, Glu83, and Asn84), in PL binding site, recorded a score of − 5.4529 kcal/mol. The superimposition of the co-crystallized (red) and the docking pose (green) of BOG in PL binding site was with an RMSD of 2.0834 A° (Fig. 7).

We used the validated setup for predicting the mode of interaction of kojic acid, and orlistat (binding ligands) to PL binding site (receptor). The activity of kojic acid is attributed to its interaction with the key amino acids in PL binding site and having comparable docking pattern and scores to the co-crystallized ligand. The results are summarized in Table 3, and Fig. 6-b and c).

**Table 3:** Docking results of kojic acid and orlistat with the human pancreatic lipase enzyme

Compound	S (kcal/mol)	Amino acids	Interacting groups	Type of interaction	Length
**Kojic acid**	−3.6920	Lys80	OH	H-bond (acceptor)	3.12
		Asn84	O (C=O)	H-bond (acceptor)	2.99
		Trp252	Pyranone	Arene-Arene	3.50
**Orlistat**	−6.3694	Lys80	O (lactone	H-bond (acceptor)	3.12
		Asn84	C=O)	H-bond (acceptor)	3.08
		Trp252	O (ester C=O) CH	Arene-H	4.04

### Half maximal inhibitory concentration (IC_50_)

PL inhibitory activity of different concentrations (1, 5, 10, 25, 50, and 100 μg/ml) of the dried 100% methanolic fraction of AspsarO, kojic acid, and orlistat was determined. The IC_50_ of the dried 100% methanolic fraction of AspsarO, kojic acid and orlistat was 7.48 μg/ml, 6.62 μg/ml, and 3.72 μg/ml, respectively.

### Evaluation of the PL inhibitory activity of 100% methanolic fraction of AspsarO in a high-fat diet induced obesity animal model

Increased single oral doses, of the dried 100% methanolic fraction, of AspsarO up to 1000 mg/kg were administered to Sprague–Dawley rats. No unusual changes in behaviour, locomotor activity, or signs of intoxication, and mortality were observed, during the 28-day study period, showing the safety of the tested agent.

**Fig. 8:**
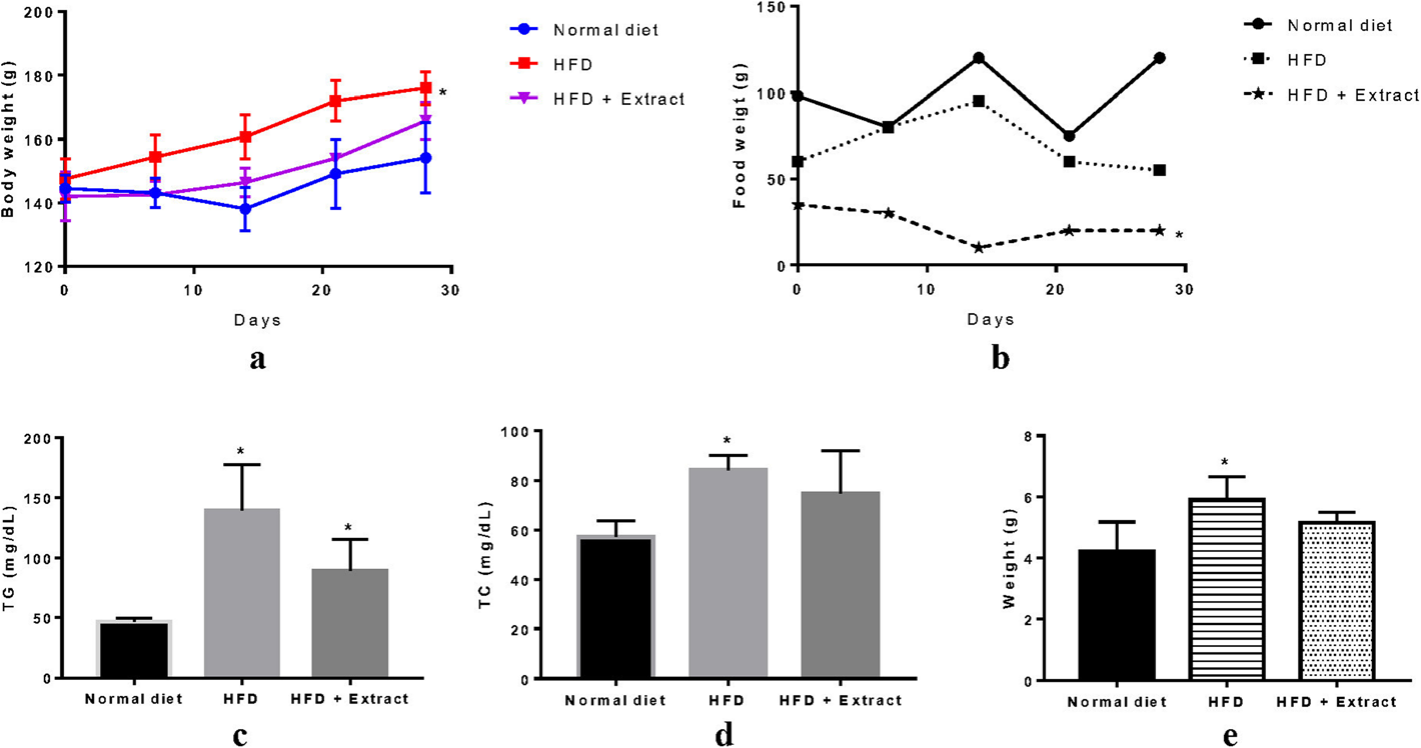
Effects of 100% methanolic extract of AspsarO on a HFD obesity-induced animal model where: **a** Average body weight vs. time, **b** Food consumption, **c** Serum triglyceride levels (TG) levels, **d** Serum total cholesterol (TC) levels and **e** Liver weight, of rats on a normal diet, HFD-fed and AspsarO treated HFD-fed rats for 4 weeks (one-way ANOVA, Holm-Sidak post hoc test, *n =* 5 per group)

Administration of a high-fat diet (HFD) to Sprague–Dawley rats, for 28 days, resulted in a significant increase in the percentage weight gain in the HFD-fed group (19.2%) compared to the control group on a normal diet (6.7%) (*p* < 0.05) (Fig. 8-a). The tested extract (100 mg/kg) caused a slight decrease in weight gain in the HFD-fed treated group (16.7%) compared to the HFD-fed control group (19.2%). Throughout the study, rats on a HFD consumed significantly less food than those on a normal diet (*p* < 0.05). Administration of AspsarO extract significantly reduced the food intake by the HFD-fed treated group compared to HFD-fed control group (*p* < 0.05) (Fig. 8-b). AspsarO extract has an appetite suppressant effect that could contribute to its anti-obesity action.

A high-fat diet caused a significant increase in serum triglycerides (TG), and total cholesterol (TC) in the HFD-fed group (*p* < 0.05). The augmented TG levels were significantly lower in AspsarO HFD-fed treated compared to the HFD-fed control group (*p* < 0.05). The TC levels were slightly lower in AspsarO HFD-fed treated compared to the HFD-fed control group (Fig. 8-c, and d). Administration of a HFD for 4 weeks resulted in a significant increase in rats’ liver weight (*p* < 0.05), while treatment with AspsarO extract resulted in a much lower increase in liver weight compared to the HFD-fed control rats (Fig. 8-e).

## Discussion

Obesity is a global health concern affecting both developing and developed countries [5]. It is correlated with the leading causes of death worldwide [11]. PL inhibitors can suppress weight gain in obese subjects [23]. Egyptian soil fungi are an under-explored resource for screening potential PL inhibitors. In this study, soil samples were collected and tested for lipase inhibition activity. PL inhibitor producers were isolated, identified, and tested in an induced obesity animal model. The active extract was further subjected to fractionation, purification, and characterization to determine the chemical structure of the active metabolite.

Thirty-nine soil samples were collected from different areas in Egypt; 38.5% of the samples showed PL inhibition activity, 66.67% of which were agriculture soils. This is in accordance with previous studies from India that reported the isolation of PL inhibitor producers from field soils [24, 25]. In our study, the active PL inhibitor producer (AspsarO) was identified as *Aspergillus oryzae* based on its colony morphology, microscopical characteristics together with *18S* rDNA sequencing. Despite the availability of molecular methods, that are advancing the identification of *Aspergillus*, the morphological method remains the most used tool for identification [26].

The quantitative lipase colorimetric assay, to measure PL inhibitory activity, was used to determine the IC_50_, which was 7.48 μg/ml in case of the 100% methanolic fraction of AspsarO extract compared to orlistat (3.72 μg/ml). The 100% methanolic fraction of AspsarO was further purified by bio-guided chromatographic techniques. The active metabolite was analyzed by ^1^H and ^13^C NMR, to confirm its structure, and was identified as kojic acid (IC_50_ of 6.62 μg/ml). Several studies reported the production of kojic acid by *Aspergillus oryzae* [20] and other soil fungi [27]. Docking studies revealed the ability of kojic acid to interact with the key amino acids in the binding site of PL similarly to the well-known standard inhibitor (orlistat) and this supports its activity. Ruilin and coworkers used the same docking approach to suggest the PL inhibitory effect of the decapeptide PP1 (Leu-Leu-Val-Val-Try-Pro-Trp-Thr-Gln-Arg) using orlistat and simvastatin as positive controls [23].

Several studies reported fungal PL inhibitors, including vibralactone isolated from *Boreostereum vibrans* [28]. The endophytic extracts from *Viola odorata* exhibited good to moderate (IC_50_ < 10 μg/ml to 10–20 μg/ml) PL inhibitory activity. VOLF4 (*Aspergillus* sp.), VOLF5 (*Peniophora* sp.), and VOR5 (*Fusarium nematophilum*) extracts exhibited PL inhibitory activity with an IC_50_ of 3.80 μg/ml, 5.85 μg/ml, and 6.52 μg/ml, respectively [8]. The ethyl acetate extract of the endophytes #57TBBALM (*Penicillium* sp.), and #AMLWLS (*Fusarium* sp.) isolated from *Aegle marmelos*, and *Taxus baccata*, respectively, had good PL inhibitory activity (IC_50_ 3.69, and 2.12 μg/ml) [7, 12].

A multi-factorial, Plackett-Burman, design allows performing a minimum collection of experimental runs, to screen for significant factors. A linear correlation between the response and a variable is sufficient for screening the main variables [29]. Our tested factors were, previously, reported to have an influence on kojic acid production [30]. The percentage of PL inhibition from the tested Plackett-Burman runs ranged from 25.9 to 61.4%. The regression equation was significant where the predicted R square value was close to the adjusted R square value, and this is an accurate measure of precision [31]. Based on our results, both incubation time and temperature significantly affected the response. The favorable temperature for PL inhibitor production was 37 °C, and the yield increased with time, reaching an optimum level on day six and decreased thereafter. This is comparable to a study by Azzahra and coworkers, who reported higher production of kojic acid in *A. oryzae* cultures incubated at 35 °C for 7 days [32].

Percentage PL inhibition was doubled when using 2% starch as carbon source; this agrees with a previous study that reported increased production of kojic acid, by *A. flavus* strain (S33–2), when using corn starch in its culture [33]. This is attributed to the production of amylolytic enzymes during the growth of *A. flavus* strain S33–2, which hydrolyses starch causing an increase in glucose concentration in cultures. Glucose has a six-carbon ring and acts as a precursor for kojic acid synthesis [34]. Similarly, corn starch was previously reported to be the optimum carbon source for kojic acid production by *A. oryzae*; using 10% (w/v) corn starch and yeast extract in *A. oryzae* cultures produced the highest yield [35]. In our study, supplementing cultures with tryptic soy broth resulted in an enhanced PL inhibitor production. Tryptic soy broth is a nutritious medium that provides amino acids, and other complex nitrogenous substances, besides glucose which is a source of energy. The presence of important growth factors, such as vitamins, and oligo-elements in specific nitrogen sources play an important role in enhancing kojic acid production [36]. On the contrary, yeast extract was reported to be the most favorable organic nitrogen source, for kojic acid production, compared to peptone and polypeptone [37]; others proposed peptone [33]. A previous study suggested the optimal medium, for fermentation of kojic acid using *A. oryzae,* to contain sucrose and yeast extract [32].

To develop effective anti-obesity treatments, various obesity-induced animal models are utilized to emulate obesity-like conditions in humans [38]. HFD feeding to Sprague-Dawley rats for 4 weeks resulted in obesity-like conditions with a significant increase in body weight, liver weight, and serum lipid levels. This agrees with previous studies reporting the usefulness of HFD-fed rats’ obesity-induced model [38]. Treatment with dried 100% methanolic fraction of AspsarO extract, at a daily dose of 100 mg/kg, resulted in a decrease in percentage weight gain of HFD-fed rats; suggesting an anti-obesity effect. Similarly, feeding rats a cafeteria diet (CD) for 6 weeks resulted in obesity-like conditions with increased body weight, liver weight, and serum lipids. Treatment with galangin, a PL inhibitor isolated from *Alpinia galangal* rhizomes at a dose of 50 mg/kg/d, resulted in a significant decrease in weight gain in CD-fed rats, indicating an anti-obesity effect [38].

Obvious suppression in appetite, with a significant decrease in food intake, was observed in AspsarO HFD-fed treated group, and this could account for the decreased percentage in weight gain. Previous reports highlighting the reduction in food intake and a decrease in body weight in HFD-fed mice and rats, receiving herb or plant extracts having a PL inhibitory effect, are available. This was attributed to metabolic changes, alteration in appetite-related peptides’ expression, or altered energy expenditure because of treatment [39].

In obese cases, the liver receives larger amounts of fatty acids; this increases its weight and accumulates the lipids in it due to the possibility of impairment of normal lipids’ catabolism [40]. In our study, a significant increase in liver weight was observed in the HFD-fed group compared to the group on a normal diet. Administration of AspsarO extract reduced the increase in liver weight in HFD-fed treated group. This effect is probably due to the inhibition of lipase activity. Also, a significant increase in serum lipids, such as TC and TG, was observed in HFD-fed rats. Treatment with the AspsarO extract resulted in a significant decrease in serum TG, and this suggests an anti-triglyceride effect with possible cardioprotective activity. A previous study reported a significant decrease in serum TG, TC, and LDL-C upon administering, a daily dose of 35 or 70 mg/kg, platycodin saponin (PL inhibitor) to HFD-fed Sprague–Dawley rats for 4 weeks [41]. Similarly, HFD-fed Sprague-Dawley rats receiving a mixture of an aqueous extract of *Salacia reticulata* and cyclodextrin had lower plasma triacylglycerol levels, body weight, and visceral fat mass compared to the HFD-fed control group. This is due to its polyphenolic constituents, with lipase-inhibitory activity, resulting in a decrease in lipid absorption from the small intestine [39]. The anti-obesity activity of rice *koji* molds, mostly *A. oryzae,* was evaluated in HFD obesity-induced animal models. Kojic acid and pyranonigrin-A are among the secondary metabolites of *koji* molds [42, 43]. Rice *koji* decreased weight gain [44], total adipose tissue [44], liver weight [45], serum TG levels [45] without affecting food intake [44] in rat obesity models; thus reducing the risk of arteriosclerosis [46]. This agrees with our observed anti-obesity and anti-triglyceride effects of kojic acid, but disagrees with the loss of appetite encountered in our study.

## Conclusions

An *A. oryzae* local isolate, from agriculture soil, can be used for the production of kojic acid. The results of the obesity-induced animal model, in silico molecular docking, and the lipase inhibitory assay provide scientific evidence for the promising future use of kojic acid, in obesity management and as an anti-triglyceride agent.

## Methods

### Collection of soil samples and isolation of fungal lipase inhibitor producer(s)

Thirty-nine soil samples were collected from various ecosystems in Egypt: fields, gardens, Nile river bank areas from July 2017–January 2018 (Table 1). Lipase inhibitor active producers were isolated according to the method of Naveen and coworkers with some modifications where: one-gram soil sample was mixed with 100 ml sterile distilled water with shaking at 10×g for 1 h at 28 °C. One ml supernatant was inoculated in duplicate in 50 ml starch casein broth and incubated at 28 °C for 7 days with shaking at 10×g. Following incubation, the mycelial mat was collected by centrifugation at 3000×g for 20 min at 4 °C. The mycelium was methanol extracted (1: 4) and assayed for its lipase inhibitory activity. One ml culture, from the duplicate culture, was spread on starch casein agar and incubated at 28 °C for 2 weeks. Colonies, recovered from samples having PL inhibitory activity, were further sub-cultured on starch casein agar followed by Sabouraud dextrose agar (Difco, USA) [47]. Isolated pure colonies were sub-cultured in starch casein broth for further testing their lipase inhibitor activity.

### Colorimetric pancreatic lipase (PL) inhibition assay

Pancreatic lipase (PL) inhibitory activity was measured colorimetrically using the substrate *p-*nitrophenyl palmitate (PNPP) (Sigma-Aldrich, USA), according to the method of Kordel and coworkers with slight modification where: the enzyme solution was prepared, immediately before use, by dissolving crude porcine PL type II (Sigma-Aldrich, EC 3.1.1.3, USA) in 100 mM Tris (pH 8.2) to get a concentration of 2 mg/ml (200 units/ml). Ten μL methanolic extracts of the suspected cultures were pre-incubated with 40 μL PL solution for 30 min at room temperature before the addition of 20 mM PNPP dissolved in isopropanol [48]. The volume was adjusted to 200 μL using 100 mM Tris (pH 8.2), and the absorbance was measured at 410 nm using a spectrophotometer (Agilent Technologies, USA). The assay was performed in triplicates, and the results were expressed as an average mean value. Orlistat (Marcyrl pharmaceutical industries, Egypt), a known PL inhibitor, was used as a positive control; a control without the inhibitor was tested in parallel. The percentage of PL residual activity was determined for each extract by comparing the activity with and without the tested compounds. Percentage inhibition of lipase activity was calculated using the formula:

Lipase inhibition = (A-B/A) × 100, where A is lipase activity in the absence of inhibitor, B is the lipase activity in the presence of inhibitor [49].

### Confirmatory fluorometric assay for lipase inhibition

The pancreatic lipase inhibitory effect was further confirmed using a fluorometric assay where: 6.0 mg 4-methylumbelliferyl butyrate (MUB) (Sigma-Aldrich, USA) was dissolved in 1000 μl DMSO and five-fold freshly diluted before the measurement. Ten μl 100% methanolic extract of the pure isolate*,* 90 μl PL enzyme (0.2 μg/ml), and 200 μl 50 mM phosphate buffer solution (pH 7.4) were added to a clear bottom black sides microtiter plate (Corning Incorporated, USA); 50 μl MUB solution was added before measurement. Orlistat was used as a positive control. The emitted fluorescence at 445 nm was measured after excitation at 365 nm with a fluorescence spectrophotometer (Agilent Technologies, USA). The speed of fluorescence development is directly proportional to the product formation, and subsequently to PL activity. The assay was repeated three times, and the PL activity was measured with and without the inhibitor [9].

### Macroscopic and microscopic examination, *18S* based rDNA sequencing and phylogenetic analysis of the lipase inhibitor fungal producer

Pure colonies, having lipase inhibitor activity, were sub-cultured on three culture media: potato dextrose agar (PDA) (20% potato infusion, 2% dextrose, and 2% agar); malt extract agar (MEA) (2% malt extract, and 1.2% agar) and Czapek yeast extract agar (CYA) (0.3% NaNO3, 3% sucrose, 0.1% K2HPO4, 0.05% KCl, 0.001% FeSO4 hydrated, 0.05% yeast extract, and 1.5% agar), and incubated at 25 °C for 7 days. Macro and micro-morphological characteristics were studied for identification to species level [26]. Colony morphology, color, size, and texture were examined. The microscopical characteristics: hyphae, conidiophores, and conidia were examined by the wet mount technique at 40X magnification by an Olympus microscope (Olympus Corporation, Japan) [50].

Genomic DNA extraction was performed as follows: 100–200 mg mycelium was placed in a 1.5 ml Eppendorf tube containing 100–150 μl 0.5 M NaOH, quickly macerated with a micro-pestle (no big chunks) and allowed to stand for 6–10 min. 500 μl Tris-HCl (100 mM, pH 8) was added followed by vortexing. After centrifugation for 10 min at 14,000 rpm, the supernatant was transferred to a new Eppendorf tube and stored at − 20 °C. One μl of the prepared DNA was used as a template in PCR reactions [51]. The PCR reaction was performed in a final reaction volume of 50 μl using the primers: *18S* F (5`- tgatccttcygcaggttcac- 3`) and *18S* R (5`- acctggttgatcctgccag- 3`) (Invitrogen, USA) at a concentration of 0.5 μM each [52], 0.5 mM dNTPs (Promega, USA), and 2 U Taq DNA polymerase (Promega, USA) in 1 × PCR buffer (Promega, USA) containing 1.5 mM MgCl_2_ (Promega, USA), using a Techne thermal cycler (Cole-Parmer, USA). The cycling parameters were as follows: denaturation at 95 °C for 5 min; 30 cycles each 94 °C for 30 s, 55 °C for 1 min, and 72 °C for 2 min; and a final extension at 72 °C for 10 min. The amplified product was purified using Wizard SV Gel and PCR clean up system (Promega, USA), and sequenced using ABI3730XL sequencer (Macrogen, Korea). The obtained sequence was blasted against the nucleotide database using blastn tool of the US National Centre for Biotechnology Information (NCBI) [53].

Phylogenetic analysis was performed using MEGA-X software [54]. The obtained *18S* rDNA sequence of AspsarO and the downloaded sequences of its closely related neighbors were aligned using Clustal W. MUSCLE algorithm was used for trimming and verification of the aligned sequences. Maximum Composite Likelihood was used to compute the evolutionary distances [8].

### Optimization of lipase inhibitor production by AspsarO

#### Incubation temperature and time

To determine the optimum incubation temperature, a 5 mm mycelial plug of a seven-day-old AspsarO culture was inoculated in 250 ml Erlenmeyer flasks containing 50 ml PDB and incubated at 20 °C, 25 °C, 37 °C, and 42 °C for 6 days with shaking at 5×g. On day six, the lipase inhibitory activity was measured [49]. As for the optimum incubation period, similar AspsarO cultures were incubated at 30 °C for 8 days, and the lipase inhibitor activity was daily monitored starting from day four till the end of the experiment [49].

#### Use of different carbon and nitrogen sources

The effect of different carbon sources on lipase inhibitor production was studied by replacing dextrose in the culture medium (PDB) with each of the following sugars: 2% sucrose, 2% lactose, or 2% starch. Also, the effect of supplementing AspsarO cultures with different nitrogen sources was studied by adding 1% of each of the following: yeast extract, peptone, tryptone, or tryptic soy broth to the culture medium (PDP) [55]. A 5 mm mycelial plug of a seven-day-old AspsarO culture was inoculated in 250 ml Erlenmeyer flasks containing 50 ml culture medium and incubated at 30 °C for 6 days with shaking at 5×g. The lipase inhibitor activity was measured on day six [49].

### Plackett-Burman design for optimization of PL inhibitor production

A Plackett-Burman design was used to identify the main variables influencing lipase inhibitor production by AspsarO. Four independent variables, with the possible low (−) and high (+) levels, were assessed for their significance on the inhibitor yield. The tested variables included: carbon source (2% sucrose-2% starch), nitrogen source (1% yeast extract-1% tryptic soy broth), production time (5–6 days), and temperature (25–37 °C). Table 2 shows the tested medium ingredients and incubation conditions of the 16 runs of the assessed factors besides the 17th run under control conditions. Minitab 18 software was used to generate the design and analyze the outputs of the experiments. The calculated E-value magnitude of the tested factor shows its effect or its significance in affecting the response. The positive or negative sign of the E-value is indicative of its positive or negative influence on the responses [56]. All runs were performed in triplicates, and the lipase inhibitory activity was determined [49].

### Production, purification and identification of the lipase inhibitor

To achieve maximum PL inhibitor production, AspsarO was inoculated in potato starch broth containing tryptic soy broth at 37 °C for 6 days. At the end of fermentation, the biomass was separated from eight liters cultures. The broth was subjected to fractionation using methylene chloride (3 × 300 ml). Pooled methylene chloride soluble fractions were evaporated under reduced pressure (Büchi R-100 Rotary Evaporator, Germany) to get a brownish residue (60 mg). The remaining aqueous layer was concentrated under vacuum and applied to a DiAION HP-20 column (5 × 100 cm) (Supelco Analytical**,** Germany) and eluted with water, 50, and 100% methanol. The collected fractions were dried under vacuum to obtain the dried aqueous (260 mg), 50% (800 mg), and 100% (1.02 g) methanolic fractions. Dried fractions were dissolved in DMSO, and the lipase inhibitor activity was determined. The fractions showing a lipase inhibitor activity were further analyzed by TLC using methanol and chloroform (95: 5 v/v) as a mobile phase on Silica gel 60 TLC plates (Merck, Germany) [49].

Using a bio-guided approach, the 100% methanolic fraction was further purified to isolate the main lipase inhibitor. It was chromatographed using PuriFlash 4100 (Interchim, France) using 25 g flash cartilage (silica gel 60, 30 um), and eluted with CH_2_CL_2_: methanol (9.5:0.5 ~ 90:10 v/v). Twenty ml fractions were collected, and the fractions were monitored using TLC and visualized under UV. Fractions with major spots were collected and evaporated under vacuum to obtain semi-pure fractions (300 mg). They were purified on silica gel columns (2 × 20 cm) using CH_2_Cl_2_: methanol (9.5:0.5 v/v) as eluent to get a pure compound (10 mg off-white powder). The identity of the compound was assessed using ^1^H and ^13^C NMR, and the obtained data was compared to literature [22].

### Molecular docking study

The Molecular Operating Environment (MOE, 2015.10) software was used in all molecular modeling studies. All minimizations were done, using MOE, until an RMSD gradient of 0.05 kcal∙mol^− 1^ Å^− 1^ was reached using a MMFF94x force field. Partial charges were calculated automatically. The X-ray crystallographic structure of human pancreatic lipase (PDB ID: 1LPB) was downloaded from the protein data bank [57]. We removed the water molecules and ligands not involved in binding from the co-crystallized enzyme. The enzyme was prepared, for docking, using the *Protonate 3D* protocol in MOE with default settings. The co-crystallized ligand was used to define the binding site. Triangle Matcher placement method and London dG scoring function were used in docking [58].

### Determination of IC_50_

Dried 100% methanolic fractions of AspsarO and kojic acid were dissolved in DMSO to get the following concentrations: 1, 5, 10, 25, 50, and 100 μg/ml. Similar concentrations of orlistat (reference standard) were also prepared. The PL inhibitor activity of all the prepared concentrations was measured [49]. IC_50_ was calculated by plotting log (dose)-response inhibition curve using the equation “log (inhibitor) vs. response” with GraphPad Prism software [8].

### Evaluating the AspsarO lipase inhibitor in a high fat diet (HFD) induced obesity animal model

#### Animals

Six weeks old male Sprague-Dawley rats weighing from 125 g to 165 g were purchased from New veterinary center (Cairo, Egypt). Rats were kept in the laboratory animal housing at the faculty of Pharmacy, Cairo University, following the recommendations of the guide for care and use of laboratory animals. They were randomly assigned to polycarbonate cages, with bedding of husk, and 12-h light/dark cycles; feed and water were given ad libitum. Environmental conditions were maintained at a temperature of 22 °C ± 2 °C and relative humidity of 60% ± 10%. All animal procedures were performed as per the international ethical guidelines and the National Institute of Health guide concerning the care and use of laboratory animals.

#### Acute toxicity testing

The acute toxicity test of the dried 100% methanolic fraction of AspsarO was performed as per the organization for European economic cooperation (OECD) guidelines No. 420. Increasing doses of 100, 400, 800, and 1000 mg/kg of the tested extract in distilled water, were administered as a single dose (one ml) by oral gavage to four rats; one rat received distilled water and served as a control. Pharmacotoxicity signs like changes in the skin, fur, eyes, respiratory and central nervous systems, and any changes in behavior or physical activities were observed at 10 min, 30 min, 60 min, 120 min, 4 h, and 6 h after treatment. Treated animals were daily monitored during the period of the study for mortality, and any pharmacotoxicity signs [59].

#### Experimental design

Following 1 week of acclimatization with pelletized commercial diet, we randomly divided the rats into three groups of five rats each. The groups were as follows: group on a normal diet, a HFD-fed group, and a HFD-fed group receiving 100 mg/kg/day of the dried 100% methanolic fraction of AspsarO dissolved in water. The extract was administered as a single daily oral dose for 28 days. HFD was prepared by mixing 35% ghee with the ground standard diet. The food intake and body weight were monitored every 48 h [60].

#### Biochemical parameters

Following 4 weeks of treatment, blood samples were collected from 12 h fasted rats by retro-orbital puncture, and serum was separated by centrifugation at 2000×g for 10 min. Serum total cholesterol (TC) and triglycerides (TGs) were determined using commercial kits (Roche, Germany) and Cobas 8000 automated analyzer (Roche, Germany). Rats were sacrificed by cervical dislocation under anesthesia, and the livers were dissected and weighed [38, 59].

### Statistical analysis

We used the GraphPad Prism 7 software for statistical analysis, including t-test, One-way ANOVA, and Dunnett’s multiple comparisons post-test at a *p*-value < 0.05. Results were expressed as mean ± standard deviation (SD) (*n* = 3). Also, unpaired Student’s t-test and One-way ANOVA followed by Sidak’s or Holm-Sidak’s multiple comparisons tests were used for analyzing the animal model experiments. The results were expressed as mean ± standard error (SEM) (*n* = 5) and were considered significantly different at a *p*-value < 0.05 (GraphPad Prism, version 7, GraphPad, La Jolla, CA).

## Supplementary information

**Additional file 1: Figure S1.** AspsarO colonies morphology on PDA, CYA and MEA where: (a, c, e) are AspsarO front view on PDA, CYA and MEA, respectively; (b, d, f) are AspsarO colonies reverse view on PDA, CYA and MEA, respectively.

**Additional file 2: Figure S2.** AspsarO microscopical characters where: a) Wet mount showing AspsarO conidia head under Olympus microscope at 40X magnification. Conidia heads are radiate spherical to globose. b) Conidia spores of AspsarO under Olympus microscope at 40X magnification. Conidia spores are round to oval, and arranged in chains.

## Data Availability

The datasets used and/or analyzed during the current study are available from the corresponding author on reasonable request.

## Abbreviations

CD: Cafeteria diet
CYA: Czapek yeast extract agar
FDA: Food and drug administration
HFD: High fat diet
KA: Kojic acid
MEA: Malt extract agar
MUB: 4-methylumbelliferyl butyrate
PDA: Potato dextrose agar
PL: Pancreatic lipase
PNPP: *p-*nitrophenyl palmitate
TC: Total cholesterol
TG: Triglycerides
TSB: Tryptic soy broth
WHO: World Health Organization
YE: Yeast extract
