# Supplementary figures and images for: Kojic acid repurposing as a pancreatic lipase inhibitor and the optimization of its production from a local Aspergillus oryzae soil isolate
Source: BMC Biotechnol. 2020 Oct 2;20:52. doi: 10.1186/s12896-020-00644-9 (PMC7532584; doi:10.1186/s12896-020-00644-9)

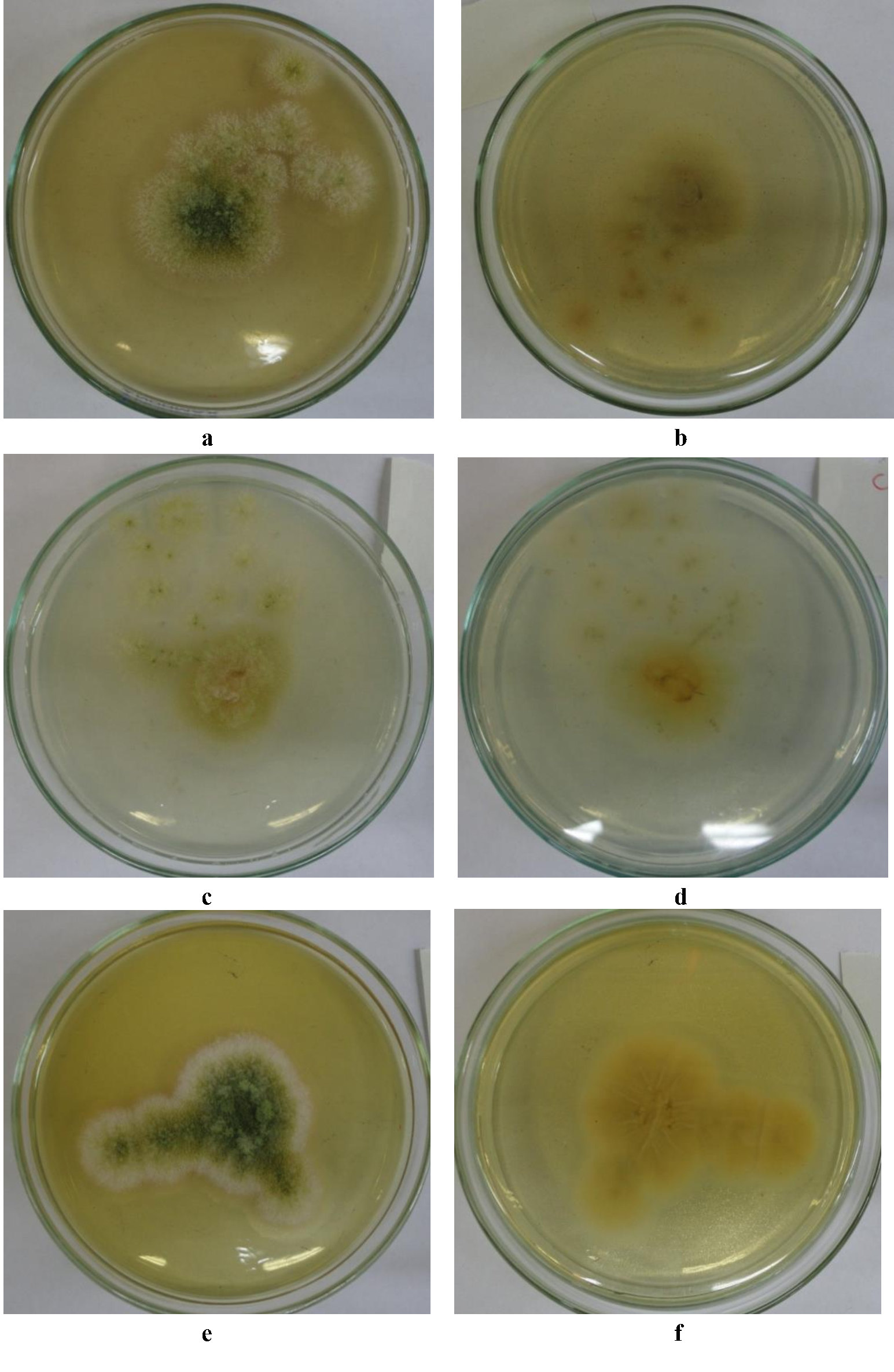

Supplement: Supplementary file 1 — Additional file 1: Figure S1. AspsarO colonies morphology on PDA, CYA and MEA where: (a, c, e) are AspsarO front view on PDA, CYA and MEA, respectively; (b, d, f) are AspsarO colonies reverse view on PDA, CYA and MEA, respectively. [file 12896_2020_644_MOESM1_ESM.jpg]

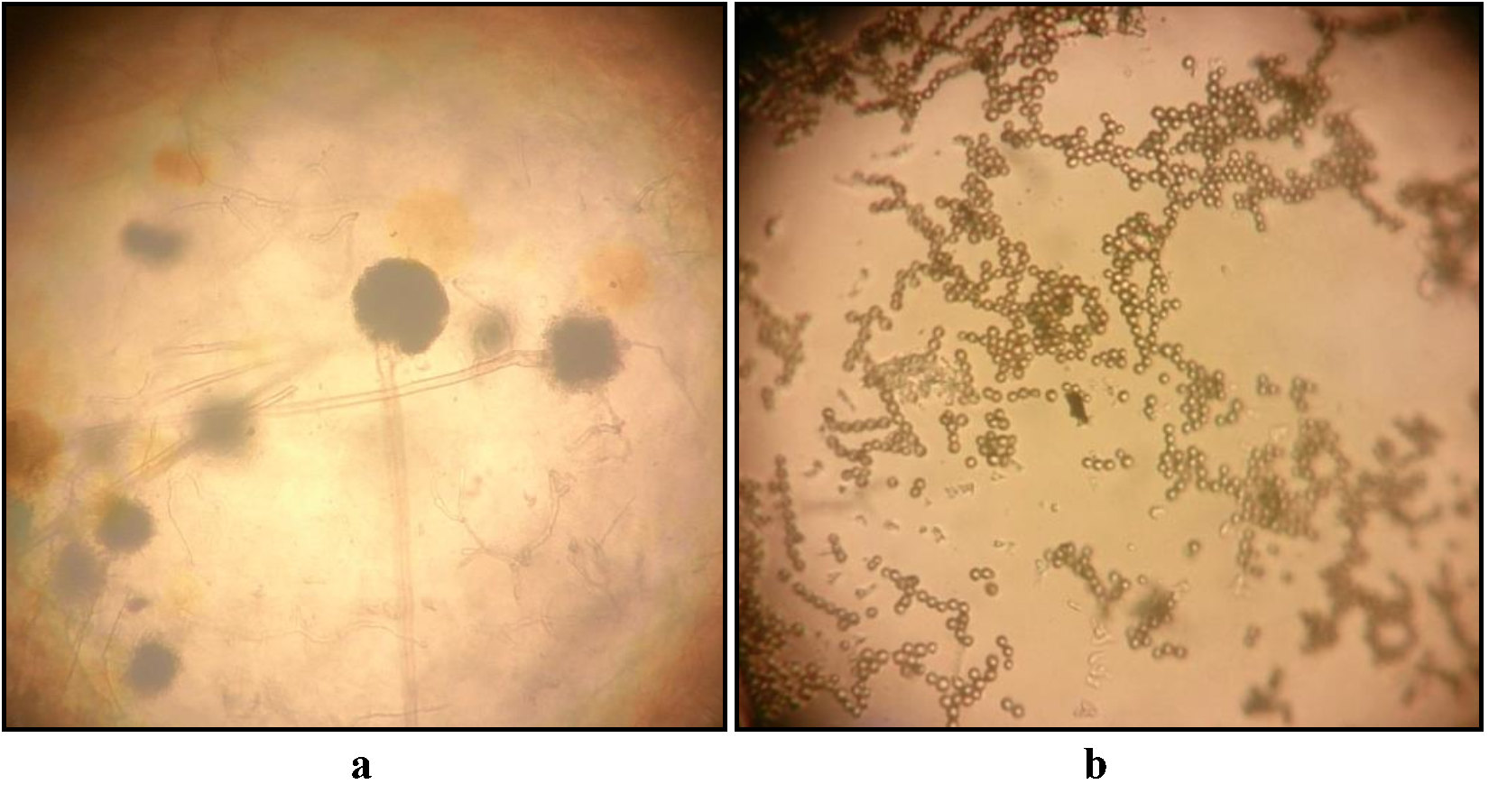

Supplement: Supplementary file 2 — Additional file 2: Figure S2. AspsarO microscopical characters where: a) Wet mount showing AspsarO conidia head under Olympus microscope at 40X magnification. Conidia heads are radiate spherical to globose. b) Conidia spores of AspsarO under Olympus microscope at 40X magnification. Conidia spores are round to oval, and arranged in chains. [file 12896_2020_644_MOESM2_ESM.jpg]
